# Implantation

**Published:** 1896-08

**Authors:** 


ARTICLE VII.

### IMPLANTATION.

Dr. Younger: So far my cases are all intact. The first case I had was a young maiden, an Italian. This, to my surprise, remained in eighteen months. All her friends came up and shook the tooth and found it had become as firm as the others.

My next case was the 17th of August succeeding. I planted five teeth, two of them on that day, the 17th, and a week later, one about the first of December, and the fifth one, a molar, in the year following. So that leaves four teeth that have been in four years, one over ten years and one ten years about the first of next August, and they are all in perfect condition. A year or two ago Dr. Daboll, of Paris, saw them, and he could not determine the teeth, or distinguish these from the original teeth.

There are quite a number of other cases that are from nine to ten years of age that are in excellent condition.

A week or so before I left, a lady visited my office and complained that the one I planted nine years ago was loose. I examined it and found what I supposed was a case of resorption of the root, so I made an appointment and prepared a tooth for a case of transplantation; When I started in to remove it I found to my surprise a solid tooth. The one that had been planted nine years last April was in perfect condition. This shows that the operation is a permanent one; that is, if an operation will last ten years, you may look on it as permanent. It promises in the case I have mentioned to last a lifetime, because there is nothing to indicate in appearance that there has been any absorption at all.

I would like to ask Dr. Jack how he makes his incision, because I think that is important in securing an artistict effect.

Dr. Jack: I generally make the incision with the circular knife.

Dr. Younger: In case there would be much absorption, that will give an unartistic appearance. I would like to show how I perform the operation to get an artistic result.

A lady went to New York for whom I planted four teeth. I sent her to a gentleman there who had performed this work a great deal and who makes a specialty of it, and I followed in three weeks. He said, "How do you do it? These teeth have been in six weeks, and I couldn't tell which of them you had planted." I said, "The thing ought to be done artistically." We want to resemble the teeth that grew there, otherwise I do not consider it artistic.

The majority of the profession look on it as a thing to be dreaded. After the tooth has been thoroughly cleansed and all the substance removed, it is incapable of carrying infection. The only bacteria we have to dread is what is known as surgical bacteria. We don't care for the tubercular or syphilitic bacteria. Before performing the operation the mouth should be thoroughly sterilized, even the tissues underneath the gum, with a delicate syringe, and the person should gargle his throat; if possible, make the territory of the operation perfectly inhospitable to pathogenic germs; then, of course, the instruments should be sterilized; the hands should be thoroughly sterilized, and the finger-nails especially, to see that there is no dirt; remove this with soap and hot water and then sterilize them. With all these precautions it is impossible for bacteria to infect the parts. Then, after the operation, every two hours the patient should sterilize her mouth, and it should be kept antiseptic as long as the wound is open. I think it is due to these precautions that I have met with so much success.

The doctor then proceeded to illustrate his operation by blackboard drawings, explaining the cuts as he made them.

Dr. Darby: Do you inject cocain into the gum or etherize the patient?

Dr. Younger: I inject cocain.

Dr. Darby: I want to bear testimony to four teeth I saw in a mouth that Dr. Younger planted at least eight years ago. There were two centrals and two bicuspids, and had been in eight years. With one of the centrals resorption had taken place, and I was forced to replant another tooth in its place. His remarks in regard to artistic appearance I can fully verify; the appearance was certainly beautiful, and there was no recession of the gums present.

Dr. Younger: A gentleman in the Confederate service, who had, in a charge, lost three centrals and a lateral, came to me for advice five years ago. He had no alveolus except at the very end of the root. In this same gentleman one of the laterals I implanted had two failures; with third I had excellent success.

I had a case where a lady came to me to have a tooth implanted, a right superior bicuspid; this was nine years ago, and I defy any dentist to select that tooth by observation or by resonance. She had one tooth out, the second superior bicuspid, and another one was diseased at the root, and she decided to have that one out and one implanted, and I performed this operation four times before I succeeded.

Dr. Darby: What do you think is the form of attachment between the tooth and the alveolus?

Dr. Younger: I think there is a great deal of what I understand to be physiological tissue and pericementum.

The doctor was asked, How long do you leave your appliances on before you consider it a success or failure?

Dr. Younger: In the course of a month or two months, if the tooth does not take hold, I take it out and put in another. If there is no attachment, of course it is a failure; if there is attachment to the gum and the tooth simply remains loose, I consider that a success.

Dr. Deane: I want to bear testimony to a few of the cases that Dr. Jack kindly invited me to his office to see, and one point that struck me is his method of retaining the tooth in the mouth. It would seem almost impossible for him to fail with the retaining fixture in place. I saw one where he had just removed the fixture, and it was impossible to produce any movement of the tooth, and that was one in which considerable pyorrhea had existed. An additional cause of trouble was that pyorrhea extended throughout the mouth, and yet this root was immovable and thoroughly resonant; I could not tell which root had been implanted.

Dr. Quillian: Dr. Jack kindly sent one of his patients over to me, and I was delighted with the result; particularly one where there had been considerable pyorrhea.

Dr. Jack: When I secure suitable teeth, I preserve them in a mixture of glycerin and water, equal parts, to keep them moist.—*International.*

